# Knowledge, Attitude, and Practice of Contraception Use Among Childbearing Women in Makkah Region, Saudi Arabia

**DOI:** 10.7759/cureus.34848

**Published:** 2023-02-10

**Authors:** Sarah S Alsharif, Rowaina I Abu Saeed, Raneem F Alskhairi, Safwan A Almuwallad, Fatimah A Mandili, Mokhtar Shatla

**Affiliations:** 1 College of Medicine, Umm Al-Qura University, Makkah, SAU; 2 Anesthesiology, King Abdulaziz Medical City, Jeddah, SAU; 3 Nursing, Umm Al-Qura University, Makkah, SAU; 4 Family Medicine, Umm Al-Qura University, Makkah, SAU

**Keywords:** saudi arabia, contraceptive use, family planning, contraception, practice, attitude, knowledge

## Abstract

Introduction

The rapid growth of the Saudi Arabian economy led to socio-demographic changes, with an increasing need for birth spacing and the use of contraceptives. This study evaluated the knowledge, attitude, and practice of contraception use in the Makkah region of Saudi Arabia.

Methods

This cross-sectional study used an online questionnaire involving women aged 18-49 in Makkah, Saudi Arabia. Descriptive analyses were performed and the Chi-squared test was used to compare variables. A p-value of < 0.05 was considered statistically significant.

Results

The study included 352 women aged 32±9.1 years with a response rate of 91%. Among them, 72.1% had a diploma or bachelor's degree, and two-thirds were married (63.4%). Nearly all respondents had heard of contraception before (96.9%). However, only 44.3% knew contraception is a method of family planning, and 23.9% knew it prevents unwanted pregnancy. During the period of conducting the research, 37.8% reported using some contraception. Oral contraceptive pills (OCP) (97.2%, 33.8%), intrauterine devices (IUD) (97.2%, 22.7%), and male condoms (92.9%, 16.9%) were the most known and popular contraception methods used. Respondents' primary sources of information regarding contraception were relatives or friends (38.3%) and websites (30.2%), and 61.9% needed education on family planning. We found that women with multiple children were significantly more likely to practice family planning (p=0.005).

Conclusion

We found that participants were aware of and had a good attitude toward family planning. However, they had poor knowledge and poor practice of family planning. Raising awareness and education are recommended to improve knowledge and practice of family planning.

## Introduction

The overall fertility and birth rate in Saudi Arabia are significantly greater than those in Western nations. However, the birth rate has fallen significantly recently, from 26.91 in 2000 to 17.09 in 2020 [1-3].

The socio-demographics of the Saudi Arabian community are rapidly changing, especially those affecting women's employment and education. This is a key factor in the shift in attitudes and practices surrounding fertility, which has increased birth spacing and, as a result, the use of contraceptives [1].

In the Kingdom of Saudi Arabia, there are several methods of contraception, with oral contraceptives being the most popular and then intrauterine devices (IUD) [3-6]. However, some studies showed that barrier contraceptives, such as condom use, were the second most commonly used instead of IUDs [7,8]. In addition, contraceptives can be used for purposes other than family planning and preventing unwanted pregnancies, such as managing menstrual disorders, reducing the risk of ovarian and endometrial tumors, symptomatic treatment for polycystic ovarian syndrome (PCOS), and preventing sexually transmitted diseases (STDs) [9-11]. 

A Saudi study conducted in the central region showed that 66.1% of women know little or wrong information about family planning, which is the opposite of Indian and Sudanese studies showing that 98% and 87%, respectively, had good knowledge. Also, previous Saudi, Indian, and Sudanese studies showed that the participants had a positive attitude towards family planning: 95.2%, 71%, and 72.5%, respectively [12-14]. Therefore, this study aimed to evaluate the knowledge, attitude, and practice of contraception use in the Makkah region of Saudi Arabia.

## Materials and methods

Study design

A cross-sectional study was conducted from the 28th of September to the 6th of October 2021 and included Saudi women aged 18 to 49 living in the Makkah region of Saudi Arabia. The sample size was calculated using OpenEpi version 3.0 (www.openepi.com) considering the following: the population size of 15-49 years old Saudi females living in Makkah is about 1,266,128, according to the General Authority for the Statistics of the Kingdoms of Saudi Arabia [15]. Assuming 50% of women knew about family planning, the minimum sample size to achieve the confidence interval (CI) level of 95% and 5% margin of error was 385.

Instrument

We used an online questionnaire distributed via social media apps for data collection. The questionnaire covered the following areas: socio-demographic data, general knowledge, attitude, and practice toward family planning consisting of open and multiple-choice questions, with single or multiple answers required. The questionnaire was developed from similar studies [1, 3, 6,12]. A family medicine consultant evaluated and modified the questionnaire for face and content validity and tested the study on 15 interested participants. The test's results have been used to improve the clarity of the questionnaire and determine the time required to complete it.

The questionnaire was anonymous, and no personal identification data needed to be collected. The initial English questionnaire was translated into Arabic (the native language of participants) and then translated back to English for publication.

Statistical analysis

Data were analyzed by the Statistical Package for Social Sciences (SPSS®) software for Windows, version 25.0 (IBM Corp., Armonk, USA). Numerical variables were presented as mean and standard deviation. For categorical variables, frequency and percentages were used. For the comparison of variables, the Chi-squared test was used. A p-value of < 0.05 was considered statistically significant.

Ethical statement

The study was approved by the Medical Ethics Committee of the faculty of medicine at Umm Al-Qura University (Ref. No: HAPO-02-K-012-2021-09-733). Electronic informed consent was obtained from each participant to submit their answers after informing them about the aim and benefits of the study and that they could withdraw at any time.

## Results

A total of 352 responses (91%) were received and respondents were mainly from Makkah city 286 (81.3%) and Jeddah 47 (13.4%). Their age ± mean was 32 ± 9.1 years. The majority, 243 (72.1%), had a diploma or bachelor's degree, and 140 (39.8%) were housewives with an income of less than 5,000 SR (Saudi riyal). Regarding marital status, almost two-thirds, 223 (63.4%), were married, and 136 (38.6%) women had two to four children. The reported cases of previous abortion were 107 (30.4%) (Table 1).

**Table 1:** Socio-demographic characteristics of study participants

Variable	Category	Frequency (%)
Age (Mean ± SD years)	32 ± 9.1	
Residency	Makkah	286 (81.3)
	Jeddah	47 (13.4)
	Taif	19 (5.4)
Marital status	Single	109 (31.0)
	Married	223 (63.4)
	Divorced or widowed	20 (5.7)
Education level	High school and less	79 (23.4)
	Diploma and Bachelor	243 (72.1)
	Master and PhD	15 (4.5)
Income (Saudi Riyal)	Less than 5K	178 (50.6)
	5-10K	68 (19.3)
	11 -15K	43 (12.2)
	More than 15K	21 (6.0)
	Prefer not to answer	42 (11.9)
Job	Student	104 (29.5)
	Employed	74 (21.0)
	Unemployed	28 (8)
	Retired	6 (1.7)
	Housewife	140 (39.8)
Number of children	0	135 (38.4)
	1	29 (8.2)
	2-4	136 (38.6)
	5 and above	52 (14.8)
Previous history of abortion	No	245 (69.6)
	Yes	107 (30.4)

Nearly all respondents, 341 (96.9%), had heard of contraception before. When asked about the meaning of contraception, their responses were: 156 (44.3%) responded that it is a method of family planning, 84 (23.9%) said it is a way to prevent unwanted pregnancy, and only four (1.1%) thought it could protect from sexually transmitted diseases, while 103 (29.3%) responded by choosing all of the above definitions. We found that the most known type of contraception was oral contraceptive pills (OCP), followed by intrauterine devices (IUD) and male condoms by 342 (97.2%), 327 (92.9%), and 311 (88.4%) respondents, respectively. On the other hand, the least known type of contraception was a contraceptive sponge, cervical cap, and female condom, by 331 (94%), 314 (89.2%), and 257 (73%) respondents, respectively. Furthermore, most mentioned that their main source of information regarding contraception was from relatives or friends, 240 (38.3%), and websites, 189 (30.2%). More than half said they seriously needed further education about family planning 218 (61.9%) (Table 2).

**Table 2:** Response of participants to question on contraception knowledge

Questions	Frequency	%
According to your knowledge, what does contraception mean?		
Method of family planning	156	44.3
Method to prevent unwanted pregnancy	84	23.9
Could protect against sexual disease	4	1.1
All of the above	103	29.3
Do not know	5	1.4
Have you heard about contraception?		
No	11	3.1
Yes	341	96.9
What type of contraception do you know of?		
Male condom		
Do not know	41	11.6
Know	311	88.4
Oral contraceptive pills (OCP)		
Do not know	10	2.8
Know	342	97.2
Intrauterine device (IUD)		
Do not know	25	7.1
Know	327	92.9
Contraceptive implant		
Do not know	118	33.5
Know	234	66.5
Emergency contraception Pill		
Do not know	196	55.7
Know	156	44.3
Contraceptive ring		
Do not know	227	64.5
Know	125	35.5
Cervical cap		
Do not know	314	89.2
Know	38	10.8
Injectable Contraceptive		
Do not know	162	46
Know	190	54
Female condom		
Do not know	257	73
Know	95	27
Sterilization (vasectomy or fallopian ligation)		
Do not know	213	60.5
Know	139	39.5
Contraceptive patch		
Do not know	89	25.3
Know	263	74.7
Contraceptive sponge		
Do not know	331	94
Know	21	6
Source of information		
Doctor	150	24
Pharmacist	37	5.9
Websites	189	30.2
Relatives and friends	240	38.3
TV or Movies	1	0.2
Courses and studies	9	1.4
Do you need more education regarding family planning?		
No	134	38.1
Yes	218	61.9

**Figure 1:**
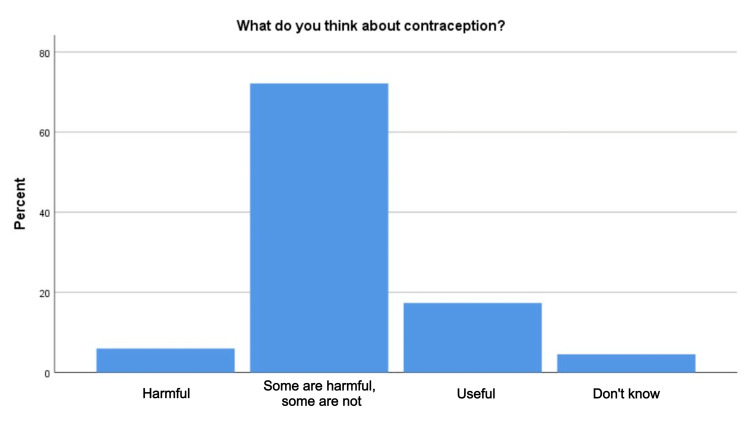
Participants' attitudes toward contraception

Regarding the aspect of attitude and practice, more than 60% believed that some contraception methods are useful, and some are not. Only 133 (37.8%) respondents were using some type of contraception during the study period. Nearly 93.2% of those on contraception reported prevention of unplanned pregnancy as their reasons and the most popular type of contraception used was OCP, IUD, and male condoms (33.8%, 22.7%, and 16.9%, respectively). The rest (7%) were using contraception for other health issues. There was a total of 20 (15%) cases of reported pregnancies while using some type of contraception method. Of those pregnancies on contraception, six (30%) were on IUD, five (25%) on a male condom, and four (20%) occurred while using natural family planning (Figure 1 and Table 3).

**Table 3:** Participants’ practice of contraception ^† ^21 participants use more than one types IUD: intrauterine device

Questions	Frequency	%
Do you use contraception?		
No	219	62.2
Yes	133	37.8
Reasoning for using contraception (N=133)		
Prevent unplanned pregnancy	124	93.2
Prevent pregnancy due to health problems	7	5.3
Regulate menstrual cycle	2	1.5
The type of contraception used† (N=133)		
Oral contraceptive pills	52	33.8
Natural Family planning	23	14.9
IUD	35	22.7
Male Condom	26	16.9
Contraceptive implant	8	5.2
Contraceptive patch	6	3.9
Fallopian ligation	1	0.6
Withdrawal method	3	1.9
Have ever get pregnancy while use contraception? (N=133)		
No	113	85
Yes	20	15
If yes, what was the method? (N=20)		
Oral contraception pill	3	15
Natural family planning	4	20
Intrauterine device	6	30
Male condom	5	25
Contraceptive Ring	1	5
Withdrawal method	1	5

When assessing the association between using contraception and the respondents’ socio-demographic data, we found a significant positive correlation between the number of children and the use of contraception (p=0.005), especially among women who had five or more children. The correlations between contraception use and other demographic parameters were not statically significant (p>0.05) (Table 4).

**Table 4:** Association between demographic data and contraception use

Variable		Use of contraception			p-value
		No (%)		Yes (%)	
Age					
	18-25	13 (46.4)		15 (53.6)	0.88
	26-35	39 (41.1)		56 (58.9)	
	36-49	42 (42)		58 (58)	
Job					
	Student	7 (38.9)		11 (61.1)	0.10
	Employed	25 (48.1)		27 (51.9)	
	Unemployed	9 (56.3)		7 (43.8)	
	Retired	5 (83.3)		1 (16.7)	
	Housewife	48 (36.6)		83 (63.4)	
Residency					
	Makkah	71 (42)		98 (58)	0.98
	Jeddah	15 (41.7)		21 (58.3)	
	Taif	8 (44.4)		10 (55.6)	
Education					
	High school and less	18 (36.7)		31 (63.3)	0.68
	Diploma, Bachelor	71 (43.6)		92 (56.4)	
	Master, PhD	5 (45.5)		6 (54.5)	
Income (Saudi Riyal)					
	Less than 5K	34 (41.5)		48 (58.5)	0.42
	5-10K	24 (44.4)		30 (55.6)	
	More than 10K	20 (55.6)		16 (44.4)	
	More than 15K	5 (33.3)		10 (66.7)	
Previous history of abortion					
	No	57 (45.2)		69 (54.8)	0.29
	Yes	37 (38.1)		60 (61.9)	
Number of children					
	0	18 (75)		6 (25)	0.005
	1	8(38.1)		13 (61.9)	
	2-4	52 (40.6)		76 (59.4)	
	≥ 5	16 (32)		34 (68)	

## Discussion

This study aimed to evaluate Saudi females' knowledge, attitudes, and family planning practices in the Makkah region of Saudi Arabia. Results revealed that almost all participants had heard about contraception. However, only one-quarter of them could correctly define contraception. These results align with previous studies in which most women knew about contraceptives but had insufficient knowledge of the details [1,12,15-18]. Our study showed that OCP was the most known type of contraception, followed by IUD and male condoms, which agrees with other studies previously conducted in Saudi Arabia [3,6,18].

Similarly, a study conducted in Qatar reported that the most commonly known methods were OCPs (90.0%) and IUDs (89.1%), which was consistent with our findings [19]. OCP popularity could be because women look for effective and convenient contraceptives, contrary to other methods whose insertion is invasive (e.g., IUD, implants) or whose success is not controlled solely by women (e.g., condoms). When properly taken, OCP reaches a 99% effectiveness rate [20]. Pregnancy on contraception indicated by our findings was mainly on IUDs and condoms (second and third most commonly known by respondents), which might be another reason women prefer OCP over others. In Qatar, pregnancy on contraception was reported among 1.2% of women using contraception [19], while in Ethiopia, 51% of contraception first users reported pregnancy on contraception [21], which could be due to inappropriate use and unfamiliarity. However, this highlights the importance of education on contraception and proper information from professionals.

In our study, the most common sources of participants' information about family planning were relatives and friends, the internet, and the doctor. Similar findings have been reported by other studies conducted in various regions of Saudi Arabia that found relatives and friends to be the main source of information [6,18,22]. In contrast, previous studies reported that the media, such as television, the internet, and newspapers, played an important role in increasing awareness of family planning [3,23]. On the other hand, some studies conducted in India, Nepal, and Ethiopia, showed that healthcare personnel was the main source of family planning information for women [16,24-26]. Saudi cultural norms and the lack of a school curriculum teaching women about contraception could be the reason for sourcing information mainly from family and friends [27].

More than half of the participants believed that some contraceptives are beneficial and some are not, which is similar to a survey conducted in Al-Medina City on female teachers, in which more than half (55.7%) of female teachers perceived some forms of contraception as effective and others ineffective [1]. Meanwhile, studies reported favorable attitudes towards contraception for the majority of Pakistani (76%) and Indian (71%) women [13,28], contrary to another Indian study showing that most men (55.5%) and women (51.5%) had an unfavorable attitude toward contraceptive methods [25]. The contradiction between these two studies could be related to their sample differences. The first study was conducted on first-year college students, while the second was on married women, mostly aged 20-30 years, with only 10% holding at least a graduate degree and 31.5% having low socioeconomic status. That implies the influence of demographic factors that lead to different results.

On the other hand, studies have also established that family planning can change demographic factors. A study conducted in Mali, Kenya and Ukraine, and Indonesia found contraceptives lead to decreased pregnancies in high-fertility settings with a huge burden of undesired pregnancies and decreased abortions in low-fertility settings [29]. Therefore, a decrease in birthrate caused by increased contraceptive use results in fewer maternal fatalities. This is supported by an Indonesian study that reported a 37.5-43.1% decrease in maternal mortality from 1970-2017 as a result of contraceptives [30]. Low abortion and pregnancy rates reduce maternal mortality by reducing the frequency of exposure to risks and reduce stillbirths and low mortality in children.

Moreover, decreased unwanted pregnancies affect marriage rates and breastfeeding habits [29,31]. Studies that explored abortion in fertility control have reported that women mostly resort to abortion as the alternative to the absence of/or insufficient family planning services [32,33]. Some old studies have advocated for the use of both abortion and contraceptive methods to control fertility to provide women with options to choose from [34]. However, recent studies contradict the arguments of old research by recommending widespread access to contraceptive methods to discourage women from resorting to abortion due to the associated dangers of induced abortion. A study conducted in Mosul, Iraq, on women aged 15-49 found that 13.5% attempted to induce an abortion at some point, either through engaging in strenuous physical activity (66.2%), the use of herbs (22.2%), or the use of pharmaceuticals (17.6%). Factors associated with low rates of abortion included using contraceptives, older age, higher education, being employed, extended families, being consanguineous and non-polygynous marriages [35]. Low abortion rates in areas with high rates of contraceptive use were also reported in Iran, Tunisia and Turkey [35-37] confirming that abortion is used as an alternative to the absence or failure of contraceptive methods. A Pakistani study suggested that failure of contraception could contribute to more abortions as its findings showed most women seeking misoprostol within days after pregnancy [38]. Due to induced abortion serious complications, including bleeding, infections, organ damage, and infertility and death [39], contraceptives are encouraged over abortion as a method of birth control, unless medically indicated.

During the study period, 37.8% of our study participants were on different types of contraception, which is slightly higher than 29.6% reported in Abha city by another study [6] but far higher than 17% reported among Indian first-year college students [13]. However, this finding indicates poor practice compared to another study showing that 88% of women practice family planning in Al-Medina city [1], which is more than twice our findings, and 75.4% in Aseer [3]. Moreover, a study from Nepal found that most (79.3%) studied women were using family planning methods [26], which is twice the percentage we found. These differences could be attributed to the study's selection criteria, as the Al-Medina city study included teachers, 59.7% of whom were between the ages of 36 and 45, whereas the Abha City study included women of a higher age group, the majority of whom had been married for more than 11 years, implying that they used contraception earlier in their lives, as 53.5% had used contraception in the past. 

There was a significant positive correlation between the number of children and the use of contraception which is expected since families with more children usually try to limit further pregnancies. Combined with our study respondents' willingness to have contraception (61.9%), these findings indicate the need for education involving men and women and to raise awareness to improve knowledge and practice of family planning in Makkah and Saudi Arabia in general. 

Our study highlighted the need for raising awareness and education for Makkah residents on family planning. However, future interventions should take into account the context of Saudi Arabia, including demographic, cultural, and religious contexts, to effectively increase the use and success of contraception. Most data on successful contraception programs are from western countries, and there have been identified differences between Eastern and Western countries, making western strategies inaccurately fit the East context. Unlike western world, studies have indicated that most families in Asia use natural methods for contraception, moreover, multiple factors such as cultural factors, poor knowledge of contraceptives and reproduction, as well as limited family planning services are major barriers to the use of modern contraception [40] aligning with barriers reported in Aljouf, Saudi Arabia [5]. Socio-demographic factors like education and employment also influence differences in contraception practices in East and West as evidence shows that Western women who tend to be more educated and employed than Eastern women also practice contraception more than their eastern peers [40,41].

Additionally, religious differences were identified as the prevalence of contraception was found to be lower in Muslim women than in Christian women [42-44]. Since most western countries are majority Christian, these religious differences should be considered when implementing contraception programs or learning from the West middle eastern countries, which are mostly Muslim, including Saudi Arabia. Moreover, sects within Islam should be considered as one study done among Muslim women in the USA revealed that Sunni women were more likely to use contraception methods in general and condoms than Shia women, while the latter were more likely to use OCPs [44]. However, local studies in Saudi Arabia and other Muslim countries are recommended to have data on these differences among Muslim women reflecting local context.

Some of our study limitations are related to its design. It was an online study that could have resulted in selection bias. A cross-sectional design is prone to a limited determination of exposure-outcome associations due to the simultaneous measurement of both exposure and outcome. These limitations may affect the generalization of our results. Therefore, studies without these limitations, such as offline interview-based longitudinal studies with randomization of samples, are recommended.

## Conclusions

We found that participants were aware of and had a good attitude toward family planning. However, they had poor knowledge and poor practice of family planning. OCP, IUD, and condoms were the most well-known methods of contraception. We found that women with multiple children were significantly more likely to practice family planning. Pregnancies were recorded on IUDs and condoms during family planning. This study’s results indicate the need for education on reproduction and family planning to improve knowledge and practice of family planning. Early education in schools, awareness campaigns, and special education programs for couples, wives, and their husbands would increase the success of family planning, reduce pregnancy on contraception, and improve family well-being and the national economy.
